# Ultrasound Treatment Combined with Rhamnolipids for Eliminating the Biofilm of *Bacillus cereus*

**DOI:** 10.3390/microorganisms12122478

**Published:** 2024-12-02

**Authors:** Ben Niu, Yiming Sun, Yongwu Niu, Shan Qiao

**Affiliations:** 1 National Engineering Research Center for Wheat & Corn Further Processing, Zhengzhou 450001, China; niuben1011@163.com (B.N.); sunyiming1015@163.com (Y.S.); qiaoshan2021@126.com (S.Q.); 2 College of Food Science and Technology, Henan University of Technology, Zhengzhou 450001, China; 3 Food Laboratory of Zhongyuan, Luohe 462300, China

**Keywords:** biofilm, rhamnolipids, ultrasonic sound, extracellular polymer, collaborative antibacterial

## Abstract

Biofilm formation by *Bacillus cereus* is a major cause of secondary food contamination, leading to significant economic losses. While rhamnolipids (RLs) have shown effectiveness against *Bacillus cereus*, their ability to remove biofilms is limited when used alone. Ultrasound (US) is a non-thermal sterilization technique that has been found to enhance the delivery of antimicrobial agents, but it is not highly effective on its own. In this study, we explored the synergistic effects of combining RLs with US for biofilm removal. The minimum biofilm inhibitory concentration (MBIC) of RLs was determined to be 32.0 mg/L. Using a concentration of 256.0 mg/L, RLs alone achieved a biofilm removal rate of 63.18%. However, when 32.0 mg/L RLs were combined with 20 min of US treatment, the removal rate increased to 62.54%. The highest biofilm removal rate of 78.67% was observed with 256.0 mg/L RLs and 60 min of US exposure. Scanning electron microscopy analysis showed that this combined treatment significantly disrupted the biofilm structure, causing bacterial deformation and the removal of extracellular polymeric substances. This synergistic approach not only inhibited bacterial metabolic activity, aggregation, and adhesion but also reduced early biofilm formation and decreased levels of extracellular polysaccharides and proteins. Furthermore, US treatment improved biofilm permeability, allowing better penetration of RLs and interaction with bacterial DNA, ultimately inhibiting DNA synthesis and secretion. The combination of RLs and US demonstrated superior biofilm removal efficacy, reduced the necessary concentration of RLs, and offers a promising strategy for controlling biofilm formation in the food industry.

## 1. Introduction

The biofilm is an extracellular polymer secreted by microorganisms, such as proteins, polysaccharides, eDNA, etc., which wraps itself around the aggregated membrane-like material [1]. Failure to clean up bacteria during food processing and production can lead to the formation of a bacterial biofilm, resulting in secondary pollution and food poisoning [2]. *Bacillus cereus*, one of the most common causes of food poisoning globally, poses a significant risk in such situations [3]. It can adhere to the surface of a variety of food machinery, pipelines, and shelters and accumulate and secrete the extracellular matrix to form a difficult-to-remove biofilm and accumulate toxins in it [4], posing a serious threat to food safety. Therefore, it is of great significance to develop efficient inhibition and removal methods for the *Bacillus cereus* biofilm.

As an anionic green biosurfactant, rhamnolipids demonstrate considerable antibacterial activity, particularly against Gram-positive bacteria [5]. Recent reports have investigated the effectiveness of RLs in inhibiting biofilm formation. The study found that a 1% (*v*/*v*) concentration of rhamnolipids achieved a biofilm clearance rate of approximately 68% against *Staphylococcus aureus* [6], and the clearance rate for *Listeria monocytogenes* biofilm formed on polystyrene surfaces was about 50% [7]. In addition, research has shown that rhamnolipids (RLs) exhibit significant antibacterial activity against both vegetative cells and spores of *Bacillus cereus* [8,9]. However, the use of RLs alone for inhibiting and removing biofilms of *Bacillus cereus* often falls short of the desired outcomes. Consequently, researchers are increasingly focusing on developing combined antibacterial strategies to enhance biofilm removal efficacy.

As a non-thermal sterilization technology, ultrasound offers several advantages, including cost-effectiveness, ease of operation, and the ability to preserve the original color, aroma, taste, texture, and nutritional value of food [10]. It shows promising potential in the field of food antibacterial applications. However, industry practitioners have observed that ultrasonic treatment alone is insufficient to completely eliminate pathogenic bacteria during food processing, and merely increasing the frequency and power of ultrasound does not achieve the desired results [11]. Additionally, ultrasound has been demonstrated to facilitate the entry of drugs, peptides, and other substances into cells [12]. To enhance the bacteriostatic efficacy, researchers have explored the use of antimicrobial agents in conjunction with ultrasound for the treatment of pathogenic bacteria [13]. Nonetheless, there have been no reports on the antimicrobial activity of RLs in conjunction with ultrasound.

RLs are recognized for their remarkable antibacterial activity against both the vegetative cells and spore forms of *Bacillus cereus*. This study initially investigates the effects of RLs on the formation and eradication of *Bacillus cereus* biofilms. It further examines the synergistic impact of RLs combined with ultrasound on biofilm removal and characterizes their effects on the biofilm microscopic structure and metabolic activity. The aim is to elucidate the underlying mechanisms of their action, providing valuable insights into the application of RLs for the prevention and control of *Bacillus cereus* biofilms.

## 2. Materials and Methods

### 2.1. Bacterial Strains and Reagents

*Bacillus cereus* CMCC(B) 63301 was obtained from Beijing Preservation Biotechnology Co., stored at −80 °C as a frozen stock, and activated in nutrient broth medium (peptone 5.0 g/L, beef paste 3.0 g/L, sodium chloride 5.0 g/L). Rhamnolipids (≥90%) were sourced from Huzhou Zijin Biotechnology Co., Ltd. (Huzhou, China). The Bradford Protein Content Assay Kit, Reactive Oxygen Species (ROS) Test Kit, and Bacterial Genomic DNA Extraction Kit were purchased from Beijing Solepol Science and Technology Co. Resazurin was acquired from Anhui Cool Bio-engineering Co. (Hefei, China). Cell culture plates were supplied by Haimen Huangjie Experimental Equipment Co. (Nantong, China). All other materials used in this study were of analytical grade.

### 2.2. Assessment and Determination of Biofilm Formation Capacity

The *Bacillus cereus* culture was grown to a stable phase, and its concentration was adjusted to approximately 1 × 10^7^ CFU/mL (OD_600_ ≈ 1.2). Bacterial suspensions were dispensed into a 96-well plate at 200 μL per well, with six replicates per group. The plate was incubated at 37 °C for 12, 24, 36, 48, and 60 h. Upon completion of the biofilm culture, the medium was removed, and the wells were washed with PBS to eliminate any planktonic bacteria. Subsequently, 200 μL of methanol was added to each well for fixation for 15 min, then discarded, and the wells were washed with PBS. Each well was then stained with 200 μL of 1% crystal violet solution for 5 min, followed by washing with PBS to remove unbound dye. After allowing the wells to air dry, biofilm-bound dye was solubilized with 200 μL of 33% glacial acetic acid at 37 °C for 30 min. The absorbance of the resulting solution was measured at 570 nm to quantify biofilm formation.

### 2.3. Determination of Rhamnolipids and Combined Ultrasound on the Elimination of Bacillus cereus Biofilm

Sterile media (50.0 mL) were added to flasks, and bacterial suspensions were inoculated at 2% (*v*/*v*). The final concentrations of RLs tested were 2.0, 4.0, 8.0, 16.0, 32.0, 64.0, 128.0, and 256.0 mg/L. The mixtures were subsequently transferred to a 96-well plate and incubated at 37 °C to allow biofilm formation. The minimum biofilm inhibition concentration (MBIC) was determined using the crystal violet staining method, defined as the lowest concentration at which the optical density (OD) was significantly reduced compared to the control group (without RLs).

The 96-well cell culture plate was inoculated with 200 μL of bacterial suspension per well and incubated at 37 °C to allow for mature biofilm formation. After biofilm maturation, the medium was removed and replaced with sterile medium containing RLs. US treatment was applied for varying durations, followed by incubation at 37 °C for 24 h. The ultrasound parameters were set to 200 W power and 40 kHz frequency. The experimental groups included the following: (1) control groups without RLs and without US; (2) RL-only treatment groups at concentrations of 2.0, 4.0, 8.0, 16.0, 32.0, 64.0, 128.0, and 256.0 mg/L; (3) US-only treatment groups with exposure times of 10, 20, 30, and 60 min; (4) RL + US combined treatment groups, which involved all possible pairings of the RL concentrations and US treatment times.

The 96-well plates were removed following the treatments. The optical density at 570 nm (OD_570_) of each sample group was measured according to the crystal violet staining method. The clearance rate of *Bacillus cereus* biofilm was calculated using the formula:

$$\mathrm{Clearance}\;\mathrm{rate}\;(\%)=\frac{{\mathrm{OD}}_{0}-{\mathrm{OD}}_{1}}{{\mathrm{OD}}_{0}}\times 100\%$$

where OD_0_ and OD_1_ are the absorbance measured at 0 h and after 24 h of static incubation, respectively.

### 2.4. Micromorphological Observations of Biological Coatings

The *Bacillus cereus* biofilms were cultivated as described. For the control group, RL treatment group, and ultrasonic treatment group, samples were prepared following the procedures outlined in Section 2.3 and incubated at 37 °C for 24 h. The final concentrations of RLs were 16.0 mg/L, 32.0 mg/L (MBIC), and 64.0 mg/L, respectively, while the sonication times were determined based on the results from Section 2.3. Subsequently, the 96-well plates were removed, the medium was discarded, and the wells were washed with sterile PBS buffer. The samples were first prepared and pre-cooled at −80 °C before being freeze-dried. Following freeze-drying, they were then sprayed with gold on a copper sheet base and placed into the sample chamber of the SEM for observation.

### 2.5. Determination of Bacterial Viability 

The *Bacillus cereus* biofilm was cultured, after which the medium was removed, and the biofilm was washed with PBS buffer. Subsequently, deionized water (2.0 mL) was added to the wells, and the biofilm was dispersed into the liquid through vigorous oscillation. The resazurin solution (10%) was then introduced to the bacterial suspension, which was incubated at 37 °C with shaking at 180 rpm in the dark for 2 h. Following incubation, the suspension was centrifuged at 10,000 rpm for 10 min at 4 °C, and the supernatant was collected for analysis. The excitation and emission wavelengths of the microplate reader were set to 560 nm and 590 nm, respectively, for detection.

### 2.6. Determination of Early Exercise Ability of Bacteria

#### 2.6.1. Determination of Bacterial Aggregation Ability

The experimental procedure followed the method described by Collado [14], with bacterial aggregation capacity expressed as a percentage. *Bacillus cereus* was cultured in nutrient broth for 24 h to reach a stable phase and then divided into two groups. One group was subjected to ultrasound, with the duration determined based on Section 2.3. Bacterial suspensions were inoculated at 2% (*v*/*v*) into nutrient broth containing RLs and incubated at 37 °C with shaking at 180 rpm for 12 h. After incubation, the cultures were shaken for 2 min, and the optical density at 600 nm (OD_600_) was measured immediately (OD_0_). Following a 2 h standing period at 37 °C, the supernatant was removed and its OD_600_ (OD_1_) was measured. The bacterial aggregation ability was calculated as:

$$\mathrm{Aggregation}\;\mathrm{ability}\;\left(\%\right)=\;\frac{{\mathrm{OD}}_{0}-{\mathrm{OD}}_{1}}{{\mathrm{OD}}_{1}}\;\times \;100\%$$

where OD_0_ and OD_1_ are the absorbance after culture for 12 h and the absorbance after standing for 2 h, respectively.

#### 2.6.2. Determination of Bacterial Clustering Motility

The cluster motility of the bacteria was assessed following Rashid’s method with necessary adjustments [15]. Plates were prepared with a semi-solid medium by adding 0.3% agar to the liquid medium. Two bacterial suspensions (10 µL) were placed at the center of each Petri dish, one of which was subjected to ultrasound with the duration determined as in Section 2.3. The plates were incubated at 37 °C for 24 h, after which observations and photographs were taken to assess motility.

### 2.7. Determination of Surface Hydrophobicity Bacteria

The surface hydrophobicity of *Bacillus cereus* was determined with slight modifications to the approach outlined by Rosenberg et al. [16]. *Bacillus cereus* was cultured in nutrient broth for 24 h to reach a stable growth phase and then divided into two equal groups, with one group subjected to ultrasound, as determined by the conditions specified in Section 2.3. The bacterial suspensions were then inoculated at a 1% (*v*/*v*) concentration into 3 mL of nutrient broth containing RLs. Following this, the toluene (500 µL) was added to the bacterial suspension, which was shaken for 3 min, and the optical density at 600 nm (OD_600_) of the lower aqueous phase was recorded as OD_0_. After allowing the mixture to stand for 1 h at 37 °C, the OD_600_ of the lower aqueous phase was measured again and recorded as OD_1_. The hydrophobicity was then calculated using the formula:

$$\mathrm{Hydrophobicity}\;\left(\%\right)\;=\frac{{\mathrm{OD}}_{0}-{\mathrm{OD}}_{1}}{{\mathrm{OD}}_{0}}\;\times \;100\%$$

where OD_0_ and OD_1_ are the measured absorbance before and after standing.

### 2.8. Determination of Extracellular Polymers of Biofilm

#### 2.8.1. Extracellular Polymeric Substance Extraction

The *Bacillus cereus* biofilm was cultivated and then divided into three groups: control, RLs, and US treatment. Each group was prepared according to Section 2.3 and incubated for 24 h at 37 °C. The final concentrations of RLs used were 16.0 mg/L, 32.0 mg/L (MBIC), and 64.0 mg/L, with the sonication duration determined based on Section 2.5. After incubation, the medium was removed, and the biofilm was rinsed with sterile PBS buffer. The biofilm was then resuspended in sterile PBS buffer, dispersed by oscillation, and centrifuged at 10,000 rpm for 10 min at 25 °C. The supernatant was subsequently collected for analysis.

#### 2.8.2. Determination of Extracellular Polysaccharides

For sample analysis, 1.0 mL of the supernatant was mixed with 1.0 mL of phenol and shaken for 30 s. Subsequently, 15.0 mL of concentrated sulfuric acid was added, and the mixture was allowed to stand in the dark for 15 min. Following this, the mixture was vortexed for 10 s and then incubated in a water bath at 25 °C for 20 min. The OD_490_ was measured using a microplate reader to determine the glucose concentration (standard curve: y = 45.631x + 0.0329, R^2^ = 0.9948)

#### 2.8.3. Determination of Extracellular Protein

The standard curve was generated using the Bradford protein concentration determination kit (standard curve: y = 1.657x + 0.6187, R^2^ = 0.9994). Pipette 50.0 μL of the supernatant and 0.5 mL of Coomassie Brilliant Blue G250 solution, mix thoroughly, and incubate at room temperature for 3–5 min. Subsequently, measure the optical density at 595 nm using a microplate reader.

#### 2.8.4. Determination of eDNA

The bacterial suspension was prepared by referring to the method in Section 2.8.1. The bacterial genomic DNA extraction kit was used to extract DNA from the bacterial suspension, and the optical density value at 260 nm was measured using a micro-ultraviolet spectrophotometer.

### 2.9. Statistical Analysis

Data processing and statistical analysis were conducted using The Origin 2022 software (Origin Lab Co., Northampton, MA, USA), and SPSS Statistics 20.0 software (IBM Co., Armonk, NY, USA) was used for variance analysis, if the difference was significant. All experiments were conducted in triplicate. All data were expressed as mean ± standard deviation (SD). *p* < 0.05 was chosen as the threshold for statistically significant differences.

## 3. Results

### 3.1. Biofilm Formation Capability of Bacillus cereus

**Figure 1:**
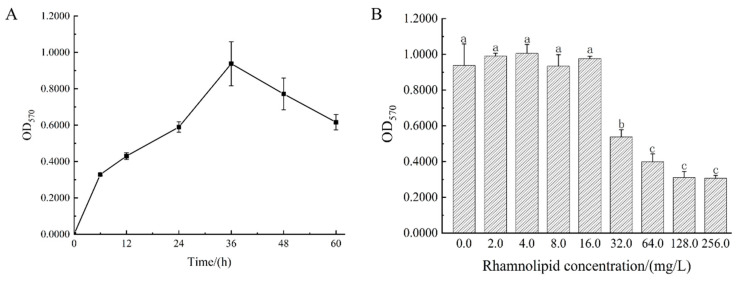
Biofilm formation of *Bacillus cereus* and inhibition of its formation by rhamnolipids. (**A**) Biofilm formation capability of *Bacillus cereus*. (**B**) Inhibition of RLs on biofilm formation of *Bacillus cereus*. The same lowercase letter means no significant difference among the relevant groups (*p* > 0.05).

The biofilm-forming capability of *Bacillus cereus* in 96-well plates was assessed. The OD_570_ values obtained through crystal violet staining indicated that biofilm formation increased initially with the duration of culture time, peaking at 0.938 ± 0.121 after 36 h (Figure 1A). This peak suggests that the biofilm was at a mature stage at this time. Following this peak, biofilm adhesion decreased, likely due to nutrient depletion and suboptimal growth conditions, which led to the release of some bacteria into the medium and a subsequent reduction in biofilm quantity. Therefore, a culture time of 36 h was chosen for biofilm formation in subsequent experiments.

### 3.2. The Minimum Biofilm Inhibitory Concentration of RLs Against Bacillus cereus

The minimum biofilm inhibitory concentration (MBIC) indicates the lowest concentration of an antimicrobial agent required to inhibit the growth and formation of bacterial biofilms. In this study, biofilm formation by *Bacillus cereus* was evaluated following a 36 h incubation period with varying concentrations of rhamnolipids. At concentrations ranging from 0 to 16.0 mg/L, no significant reduction in the formation of biofilms was observed. However, when RL concentrations were increased to between 32.0 mg/L and 256.0 mg/L, a marked decrease in biofilm formation was evident (*p* < 0.05) (Figure 1B). These results indicate that low concentrations of RLs (≤16.0 mg/L) have a minimal impact on biofilm development, while concentrations above 32.0 mg/L effectively suppress biofilm formation. Based on these findings, the MBIC of RLs for inhibiting *Bacillus cereus* biofilm formation was determined to be 32.0 mg/L.

### 3.3. Scavenging Effect of RLs on Bacillus cereus Biofilm

**Figure 2:**
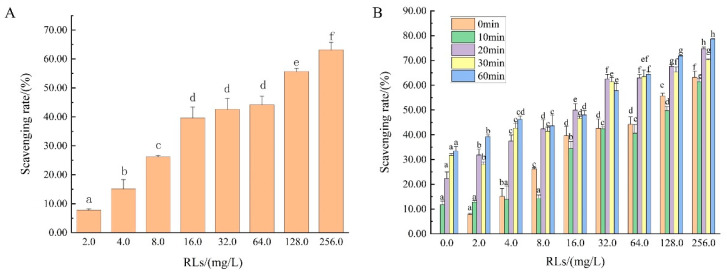
Scavenging effect of rhamnolipids alone and in combination with ultrasound on *Bacillus cereus* biofilms. (**A**) The scavenging effect of RLs on *Bacillus cereus* biofilm; (**B**) the scavenging effect of RLs and US on *Bacillus cereus* biofilm. The same lowercase letter means no significant difference among the relevant groups (*p* > 0.05).

*Bacillus cereus* was cultured for 36 h to develop a mature biofilm, and various concentrations of rhamnolipids (RLs) were subsequently applied to assess their biofilm-clearing efficacy. Compared to the control group (which had a 0% clearance rate), a concentration of 2.0 mg/L RLs demonstrated a biofilm-scavenging effect. The scavenging rate increased with the RL concentration, with 16.0 mg/L to 64.0 mg/L RLs achieving approximately 40% biofilm removal (Figure 2A). However, this increase was not directly proportional to the RL concentration. Consequently, the amount of RLs interacting with the biofilm did not increase proportionally with the concentration. At 128.0 mg/L and 256.0 mg/L, the scavenging rate improved, suggesting that RLs aggregated into smaller particles at these concentrations, which enhanced their ability to penetrate and embed within the biofilm, thus further increasing the scavenging rate. Nonetheless, after treating the *Bacillus cereus* biofilm with 256.0 mg/L RLs for 24 h, the maximum scavenging rate achieved was only 63.18%, indicating that RLs alone are insufficient for achieving efficient clearance of mature biofilms.

### 3.4. Scavenging Effect of Bacillus cereus Biofilm by RLs and US

To enhance the efficacy of biofilm scavenging in *Bacillus cereus*, combining RLs with other sterilization methods was explored. Recent advancements in non-thermal sterilization technologies, such as US, have demonstrated remarkable antibacterial properties against pathogenic microorganisms [17]. US offers significant advantages over traditional thermal sterilization methods, including the preservation of food flavor and nutrients [18]. This makes it a promising approach in food production. Research has shown that ultrasonic treatment not only exhibits substantial antibacterial activity against harmful microorganisms in nutrient-rich environments but also effectively disrupts biofilms by altering surface adhesion properties [19].

The scavenging effects of RLs and US, both individually and in combination, on the *Bacillus cereus* biofilm were assessed. When RLs were applied alone at concentrations of 16.0 mg/L, 32.0 mg/L (MBIC), and 64.0 mg/L, the biofilm scavenging rates achieved were 39.61%, 42.64%, and 44.19%, respectively. US treatment alone, applied for 20, 30 and 60 min, resulted in biofilm scavenging rates of 22.35%, 31.63%, and 33.42%, respectively (Figure 2B). These findings indicate that while both RLs and US individually demonstrate effectiveness in biofilm removal, the biofilm scavenging rates are relatively modest when each method is used in isolation.

Further analysis revealed that, with concurrent ultrasonic treatment, the biofilm scavenging rate increased with higher concentrations of RLs, indicating a positive correlation between the RL concentration and biofilm removal when combined with US. When RLs were applied at the same concentration, the biofilm scavenging rate slightly decreased with 10 min of ultrasonic treatment but significantly improved after more than 20 min (*p* < 0.05). Treatment durations of 30 and 60 min did not enhance the removal efficiency further. Considering both energy consumption and effectiveness, a 20 min ultrasonic treatment was chosen for subsequent experiments.

The combined treatment of 32.0 mg/L RLs with 20 min of US achieved a biofilm scavenging rate of 62.54%, which is comparable to the 63.18% rate obtained with 256.0 mg/L RLs alone. The maximum scavenging rate of 78.67% was achieved with 256.0 mg/L of RLs combined with 60 min of ultrasonic treatment. Based on the experimental results, it is believed that ultrasound can effectively increase the permeability of the biofilm, allowing a large amount of RLs to penetrate the biofilm. This leads to the destruction of bacterial cells, resulting in the effective removal of the biofilm.

The results indicate that combining RLs with US significantly enhances biofilm scavenging, allowing for reduced RL concentrations without compromising the removal effectiveness. The concentration of RLs has a notable impact on the scavenging rate. Based on the minimum biofilm inhibition concentration (MBIC), RL cost, and the synergistic effects with US, subsequent experiments will focus on treatment conditions, including 16.0 mg/L (1/2 MBIC) with 20 min of ultrasound, 32.0 mg/L (MBIC) with 20 min of US, and 64.0 mg/L (2 MBIC) with 20 min of US, to further investigate the microscopic mechanisms of RLs and US in biofilm removal.

### 3.5. Effects of RLs and US on the Morphology of Bacillus cereus Biofilm

**Figure 3:**
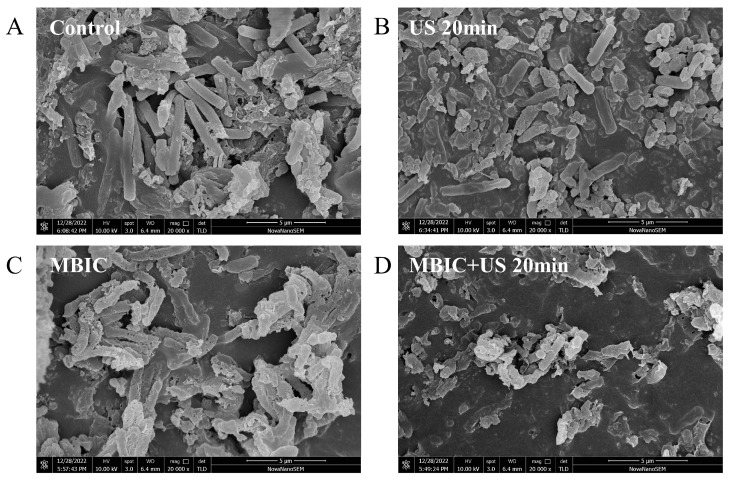
Scanning electron microscopy images of *Bacillus cereus* biofilms treated with RLs (20,000×). (**A**) No RL treatment was added, (**B**) US treatment for 20 min, (**C**) MBIC concentration of RL treatment was added, (**D**) MBIC concentration of RLs was added and treated with US for 20 min.

Scanning electron microscopy was utilized to investigate the ultrastructural changes in *Bacillus cereus* biofilms. The control group, which remained untreated, exhibited *Bacillus cereus* cells with a typical rod-shaped morphology, characterized by smooth surfaces and an intact cellular structure. After treatment with rhamnolipids at 32.0 mg/L (MBIC), noticeable morphological changes were observed, including the formation of pores and concave deformations on the bacterial cell surfaces. Treatment with ultrasound alone for 20 min resulted in a reduction in the adhesive substances between bacterial cells, leading to cracked biofilms and a more dispersed bacterial distribution. When RLs were combined with US, there was a pronounced decrease in bacterial adhesion, significant surface deformation and shrinkage of the bacterial cells, as well as a substantial reduction in the extracellular polymeric substances. These findings indicate a synergistic effect between RLs and US in disrupting both the bacterial cells and the extracellular matrix of *Bacillus cereus* biofilms, with RLs playing a predominant role in the biofilm destruction process (Figure 3).

### 3.6. Effects of RLs and US on Bacterial Metabolism in Bacillus cereus Biofilm

**Figure 4:**
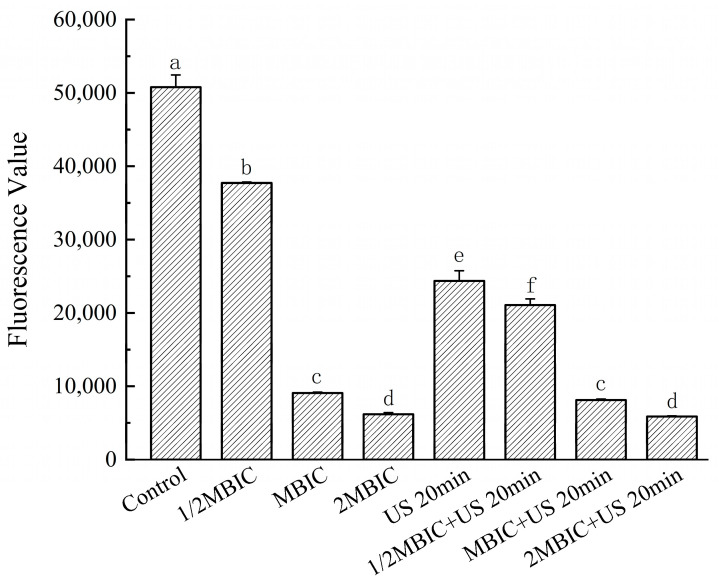
Effects of bacterial metabolism under different treatments. The same lowercase letter means no significant difference among the relevant groups (*p* > 0.05).

Metabolism encompasses the exchange of material and energy between a cell and its external environment, as well as the cell’s intrinsic self-renewal processes. The metabolic activity of bacterial biofilms, which reflects the growth and viability of the cells [18], was assessed using the resazurin assay [19]. Reduced fluorescence values correlate with decreased metabolic activity. In this study, bacterial metabolic levels were significantly reduced (*p* < 0.05) following treatment with rhamnolipids (RLs) and ultrasound (US), either individually or in combination, compared to the control group. Specifically, treatment with 16.0 mg/L, 32.0 mg/L (MBIC), and 64.0 mg/L RLs led to reductions in metabolic activity of 25.70%, 82.13%, and 87.81%, respectively. When RLs were combined with a 20 min US treatment, the metabolic reductions were 58.49%, 83.99%, and 88.42%, respectively (Figure 4). These results demonstrate that bacterial metabolic activity decreases with an increasing RL concentration, and the combined treatment of RLs and US induces more severe disruption of the biofilm metabolism. Notably, the US treatment enhanced the effects of RLs at lower concentrations (16.0 mg/L), whereas its impact became less pronounced at higher RL concentrations. This observation is further supported by scanning electron microscopy, which revealed more extensive structural damage at lower RL concentrations when combined with US.

Metabolism encompasses the material and energy exchange between a cell and its external environment, as well as the cell’s intrinsic self-renewal processes. The metabolic activity of bacterial biofilms, which reflects cell growth activity [20], was assessed using the resazurin assay [21]. Reduced fluorescence values correlate with decreased metabolic activity. In this study, bacterial metabolic levels were significantly reduced (*p* < 0.05) following treatment with rhamnolipids and ultrasound, either individually or in combination, compared to the control group. Specifically, treatment with 16.0 mg/L, 32.0 mg/L, and 64.0 mg/L RLs led to reductions in metabolic activity of 25.70%, 82.13%, and 87.81%, respectively. When RLs were combined with a 20 min US treatment, metabolic reductions were 58.49%, 83.99%, and 88.42%, respectively (Figure 4). These results demonstrate that bacterial metabolic activity decreases with increasing RL concentration, and the combined treatment of RLs and US induces more severe disruption of the biofilm metabolism. Notably, the US treatment enhanced the effects of RLs at lower concentrations (16.0 mg/L), whereas its impact became less pronounced at higher RL concentrations. This observation is further supported by scanning electron microscopy, which revealed more extensive structural damage at lower RL concentrations when combined with US.

### 3.7. The Effects of RLs and US on the Early Exercise Ability of Bacillus cereus

#### 3.7.1. The Effect of RLs Combined with US on Bacterial Aggregation Ability

**Figure 5:**
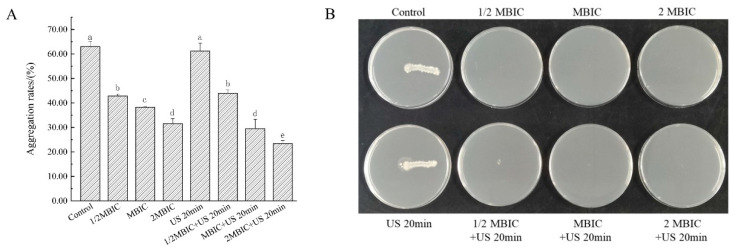
Effects of different treatments on bacterial aggregation and swarming motility. (**A**) Effects of different treatments on bacterial aggregation capacity, (**B**) effects of different treatments on bacterial swarming motility. The same lowercase letter means no significant difference among the relevant groups (*p* > 0.05).

Microbial aggregation is a crucial prerequisite for biofilm formation, and the extent of bacterial aggregation can serve as an indicator of bacterial interactions. The bacterial aggregation rate in the US medium was 63.03%. Following the addition of RLs, the aggregation rates decreased to 42.81%, 38.22%, and 31.50%, respectively, reflecting the RLs’ capacity to inhibit *Bacillus cereus* biofilm formation by disrupting bacterial interactions. Furthermore, when RLs were combined with US treatment, the aggregation rates in the US + MBIC and US + 2MBIC groups further decreased to 29.49% and 23.36%, respectively (Figure 5A), underscoring the enhanced efficacy of the combined treatment in impeding bacterial aggregation and biofilm development.

#### 3.7.2. The Effect of RLs Combined with US on Bacterial Clumping Motility

The clustering movement of bacteria, which involves rapid aggregation upon contact with an interface, is a critical behavior for biofilm formation. Both the untreated group and the US-only group exhibited strong clustering movement, indicative of their ability to aggregate. In contrast, the RL treatment group displayed a lack of clustering movement, suggesting that the US treatment for 20 min did not significantly impact bacterial viability. This observation can be attributed to the fact that the RL concentration employed reached both the minimum inhibitory concentration (MIC) and minimum bactericidal concentration (MBC) for *Bacillus cereus* in its nutritional cell state, effectively inhibiting and killing the bacteria (Figure 5B). Consequently, this inhibition of bacterial growth prevented the occurrence of clustering behavior.

### 3.8. Effects of RLs and US on the Initial Formation of Bacillus cereus Biofilm

**Figure 6:**
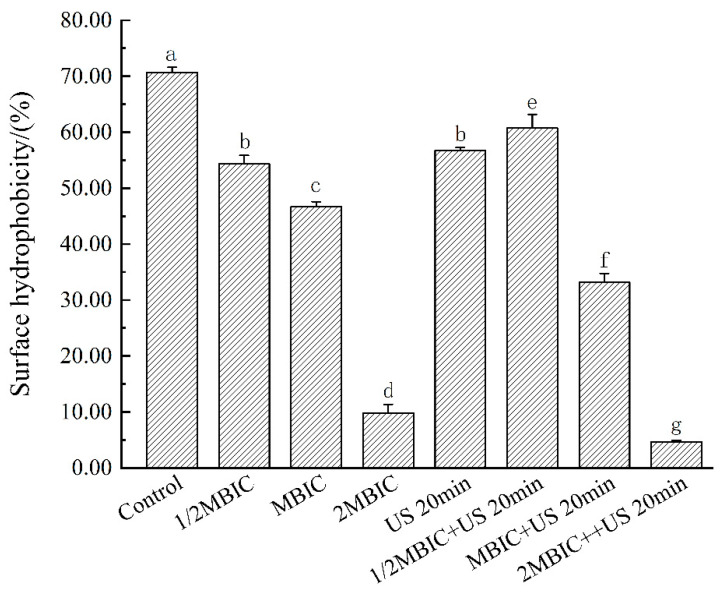
The changes of bacterial surface hydrophobicity under different treatments. The same lowercase letter means no significant difference among the relevant groups (*p* > 0.05).

Surface hydrophobicity is a crucial determinant of microbial adhesion to solid surfaces, with greater hydrophobicity correlating with increased adhesion capacity and biofilm formation [22]. In contrast, RL treatment resulted in a concentration-dependent decrease in bacterial surface hydrophobicity. At an RL concentration of 32.0 mg/L, the hydrophobicity was reduced to 46.68%. The combined treatment with US for 20 min further decreased the hydrophobicity to 33.18% (Figure 6), reflecting a significant reduction in bacterial distribution in toluene (*p* < 0.05). This combined approach effectively diminishes bacterial adhesion and inhibits the initial stages of biofilm formation.

### 3.9. Effects of RLs and US on Extracellular Polymeric Substances of Bacillus cereus Biofilm

Biofilms are complex aggregates formed by microorganisms that secrete extracellular polymeric substances under specific conditions, enveloping themselves within this matrix [23]. Extracellular polymeric substances not only mitigate stress responses induced by external environmental factors but also enhance resistance to foreign invasive agents [24]. The primary components of extracellular polymeric substances include extracellular polysaccharides, extracellular proteins, and extracellular DNA (eDNA). Extracellular polysaccharides contribute to the biofilm’s structural integrity by increasing its thickness and rigidity, thereby supporting its three-dimensional architecture [25]. Extracellular proteins play a crucial role in microbial adhesion to solid surfaces and serve as key adhesion factors that facilitate biofilm formation [26]. Additionally, eDNA is integral to bacterial adhesion, both at the interface and between cells during the initial stages of colonization [27].

**Figure 7:**
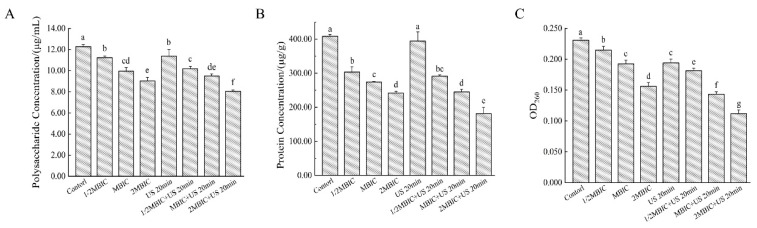
Effects of RLs and US on extracellular polymeric substances of *Bacillus cereus* biofilm. (**A**) Changes in extracellular polysaccharides of biofilms under different treatments, (**B**) changes in extracellular proteins of biofilm under different treatments, (**C**) changes in eDNA in biofilm under different treatments. The same lowercase letter means no significant difference among the relevant groups (*p* > 0.05).

The effects of various treatments on the extracellular polysaccharide content in *Bacillus cereus* biofilms were assessed. The extracellular polysaccharide concentration in the untreated control group was 12.28 μg/mL. Following treatment with RLs alone, US alone, and their combination, the extracellular polysaccharide levels in the *Bacillus cereus* biofilms decreased to varying extents. Specifically, the RL treatment at 1/2 MBIC and US alone exhibited modest inhibitory effects, resulting in extracellular polysaccharide concentrations of 11.24 μg/mL and 11.38 μg/mL, respectively (Figure 7A). In contrast, the combination treatment significantly reduced the extracellular polysaccharide content to 8.04 μg/mL.

The impact of various treatments on extracellular protein levels in *Bacillus cereus* biofilms was assessed. In untreated biofilms, the extracellular protein concentration was approximately 400 μg/g, indicating that extracellular protein is the predominant component of the extracellular matrix. Treatment with different concentrations of RLs resulted in reductions in the extracellular protein content of 25.71%, 32.81%, and 40.77%, respectively. US alone did not significantly affect extracellular protein levels (*p* < 0.05). However, combined treatment with RLs and US led to a significant reduction in extracellular protein content (*p* < 0.05), decreasing to 181.19 μg/g (Figure 7B). This suggests that the synthesis and secretion of extracellular proteins were substantially inhibited. Notably, the extracellular protein content in the US + MBIC group was comparable to that observed with MBIC treatment alone, indicating that the combined use of US and low-concentration RLs can achieve effects similar to those of higher concentrations of RLs.

The effects of various treatments on extracellular eDNA are depicted in Figure 7C. In the control group, the optical density at 260 nm (OD_260_) was 0.231. All treatments, whether applied individually or in combination, resulted in a reduction in OD values, indicating that each treatment can damage the extracellular DNA within *Bacillus cereus* biofilms. Notably, the OD_260_ values for the combined treatment groups were consistently lower than those observed with RLs alone, suggesting a synergistic effect between RLs and US. This synergistic effect is hypothesized to result from ultrasound-induced disruption of biofilm permeability, which facilitates the penetration of RLs into the biofilm matrix. This enhanced penetration likely promotes the interaction of RLs with DNA, thereby inhibiting the synthesis and secretion of extracellular DNA.

## 4. Discussion

Tsukatani et al. investigated the dynamic changes in the *Pseudomonas aeruginosa* biofilm using crystal violet staining and observed that, after 24 h of culture, the biofilm biomass gradually decreased over time. This phenomenon suggests that the formation and maintenance of biofilms may be significantly influenced by culture conditions, particularly during extended culture periods. Contributing factors may include the growth status of the bacteria, nutrient depletion, and competition with other microorganisms, all of which could lead to a reduction in biofilm density. These findings are consistent with the results of the present study [28].

Tathiane et al. demonstrated that the combination of rhamnolipids and 5% NaCl significantly and effectively reduced the planktonic bacterial population at pH 7.0, while the minimum biofilm inhibitory concentration (MBIC) decreased to 39.0 mg/L [29]. This finding indicates that rhamnolipids (RLs) possess a notable ability to inhibit the biofilm formation of *Bacillus cereus*. These results are in agreement with the existing literature, further supporting the potential application of rhamnolipids as an effective anti-biofilm agent. In contrast, this study found that the combination of rhamnolipids at a concentration of 32.0 mg/L (MBIC) with 20 min of ultrasound treatment was more effective in significantly inhibiting biofilm formation by *Bacillus cereus*, resulting in a stronger inhibitory effect.

Sanchez et al. found that when the concentration of rhamnolipids exceeds its critical micelle concentration (CMC), rhamnolipid molecules spontaneously aggregate to form micelles with distinct structural characteristics. In this aggregated state, hydrophobic groups cluster inward to form the core of the micelle, while hydrophilic groups orient outward, creating the external interface of the micelle [30]. This phenomenon partially elucidates the limitations of rhamnolipids in effectively eliminating biofilms of *Bacillus cereus* in the present study. This limitation may be attributed to the fact that beyond the CMC, the aggregated state of rhamnolipids affects its interaction with cell surfaces, thereby restricting its effective penetration and disruption of biofilms.

Ruo Park et al. investigated the synergistic effects of sodium hypochlorite and US on the bactericidal efficacy against *Bacillus cereus*. The results indicated that 50 mg/L of sodium hypochlorite, when subjected to US for 20 min, exhibited a bactericidal effect comparable to that of 100 mg/L of sodium hypochlorite applied alone [20]. In addition, Sun et al. reported that ultrasound combined with 1% lactic acid effectively eliminated free cells of *Salmonella* within 30 min. This combination was found to achieve rapid antibacterial effects against Salmonella free cells [31]. Similarly, Park et al. observed that ultrasound combined with fumaric acid significantly reduced the numbers of *Escherichia coli* and Listeria monocytogenes in apple juice [32]. These findings align closely with the results of the present study, further supporting the effectiveness of US technology in enhancing biofilm removal and microbial elimination. It is suggested that US significantly increases the permeability of biofilms, thereby facilitating greater penetration of rhamnolipid molecules into the biofilm matrix, ultimately improving the overall removal efficiency.

He Sang et al. employed scanning electron microscopy to show that gallic acid significantly inhibits bacterial adhesion and aggregation, thereby impacting the overall structure of the biofilm [33]. These findings are consistent with the results of the present study.

Lin et al. investigated the synergistic effects of a pulsed magnetic field and Lycium barbarum essential oil on *Escherichia coli* O157:H7 in vegetable juice, assessing bacterial metabolic activity using the resazurin dye method [34]. Their findings were consistent with the results of the present study. Compared to LC-EO and PMF, the synergistic treatment further reduced the metabolic capacity of bacteria by 18.07% and 7.53%. This study found through experimental research that the combination of low-concentration rhamnolipids (16 mg/L) and 20 min of ultrasound treatment significantly enhanced the inhibition of metabolic activity in *Bacillus cereus* biofilm cells, compared to the use of rhamnolipids alone without ultrasound treatment. The inhibitory effect was increased by 1.28-fold. It was clearly observed that the combined approach significantly enhanced the metabolic activity of the bacterial biofilm.

Microbial aggregation is a crucial prerequisite for biofilm formation, and the extent of bacterial aggregation serves as a significant indicator of inter-bacterial interactions [35]. Bacterial aggregation not only influences the structure and function of biofilms but also plays a vital role in their survival and stress resistance. The aggregation process involves complex interactions among bacteria, including mutual attachment, signal transmission, and cooperative behaviors. In this study, combined treatment effectively inhibited the aggregation ability of bacteria, thereby suppressing the formation, stability, and compactness of the biofilm. Microbial aggregation is a crucial prerequisite for biofilm formation, and the extent of bacterial aggregation can serve as an indicator of bacterial interactions.

Studies have demonstrated that the clustering movement of bacteria represents a rapid aggregation behavior that occurs upon contact with a surface, playing a crucial role in biofilm formation [36]. When bacteria first encounter a solid surface, those that can effectively and rapidly aggregate will form an initial adhesion layer by adhering to one another and secreting extracellular polymers. This aggregation not only enhances inter-bacterial interactions but also establishes a foundation for subsequent biofilm development. Consequently, the efficacy of biofilm elimination can be assessed by evaluating the reduction in the clustering ability of *Bacillus cereus* following combined treatment.

Koga et al. found that the removal of bacterial cell surface antigen proteins leads to a decrease in cell surface hydrophobicity, suggesting a close relationship between hydrophobicity and the bacterial surface structure. The specific immunoglobulin Y reduces the hydrophobicity of bacteria, likely by binding to surface antigen proteins, which subsequently alters the hydrophobic characteristics of the bacterial surface [37]. Husain et al. found that the total exocrine proteins, cell surface hydrophobicity, and exopolysaccharide production in *E. coli*, *L*. *monocytogenes*, *S. marcescens*, and *P. aeruginosa* experienced a significant decrease at sub-MICs [38]. This reduction in hydrophobicity may, in turn, influence the bacteria’s adhesion to host surfaces.

Zhang et al. found that ginger essential oil exhibited significant antibacterial activity against *Streptococcus suis*, with minimum inhibitory concentration (MIC) and minimum bactericidal concentration (MBC) values of 2.0 and 4.0 μL/mL, respectively. This study indicates that ginger essential oil significantly reduces the levels of intracellular ATP, nucleic acids, proteins, and extracellular polysaccharides. Moreover, ginger essential oil also affects the production of extracellular proteases, further demonstrating its ability to disrupt the membrane integrity of *Streptococcus suis* [39]. This disruption may contribute to its overall antibacterial efficacy.

## 5. Conclusions

This study investigated the efficacy of RLs alone in inhibiting and removing biofilms of *Bacillus cereus*. The minimum biofilm inhibitory concentration (MBIC) of RLs was found to be 32.0 mg/L. While RLs alone could remove biofilms, even at a high concentration of 256.0 mg/L, the removal rate was only 63.18%. To enhance the effectiveness, ultrasound (US) treatment was introduced as a secondary method. The combination of low-concentration RLs with US achieved removal efficacy comparable to that of high-concentration RLs used alone. The results indicated that the RL concentration significantly affects the biofilm removal, demonstrating that the two methods can work synergistically, with RLs being the primary contributor.

After combined treatment with RLs and US, the adhesion of *Bacillus cereus* cells was markedly reduced. The cells exhibited severe deformation and shrinkage, extracellular polymeric substances were significantly reduced, and the metabolic activity of the cells was notably diminished. The joint treatment decreased the hydrophobicity and motility of the *Bacillus cereus* cells, thereby inhibiting the initial formation of biofilms. Moreover, the combined treatment substantially inhibited the synthesis and secretion of extracellular polysaccharides, proteins, and eDNA, disrupting the formation of extracellular polymers.

This study reveals the inhibitory effect of RLs on the formation of *Bacillus cereus* biofilms but notes its limited effectiveness in removing mature biofilms. This is the first attempt to combine RLs with ultrasonic treatment for biofilm removal, demonstrating a synergistic inhibitory effect. The combined application of RLs and ultrasound offers a highly effective method for both biofilm removal and the prevention of their reformation. This synergistic approach enhances biofilm clearance from surfaces in food processing environments, improving cleaning efficiency while reducing dependence on chemical disinfectants. By promoting more sustainable sanitation practices, this method has significant potential to enhance food safety, mitigate contamination risks, and improve overall hygiene standards, positioning it as a valuable solution for the food industry.

## Data Availability

The data that support the findings of this study are available from the corresponding authors upon reasonable request.
